# A commentary on key methodological developments related to nutritional life cycle assessment (nLCA) generated throughout a 6‐year strategic scientific programme

**DOI:** 10.1002/fes3.480

**Published:** 2023-06-09

**Authors:** G. A. McAuliffe, T. Takahashi, M. R. F. Lee, A. Jebari, L. Cardenas, A. Kumar, F. Pereyra‐Goday, H. Scalabrino, A. L. Collins

**Affiliations:** ^1^ Net Zero and Resilient Farming Rothamsted Research Okehampton UK; ^2^ University of Bristol Bristol Veterinary School Langford Somerset UK; ^3^ Harper Adams University School of Sustainable Food and Farming Newport Shropshire UK; ^4^ Instituto Nacional de Investigación Agropecuaria (INIA) Treinta y Tres Uruguay; ^5^ University of Normandie ESIX Normandie Agri‐food Department Caen France

**Keywords:** environmental footprints, food systems, net zero, nutritional science, sustainability, synthesis

## Abstract

Rothamsted Research (RRes) is the world's oldest agricultural research centre, notable for the development of the first synthetic fertilizer (superphosphate) and long‐term farming experiments (LTEs) spanning over 170 years. In 2015, RRes recruited several life cycle assessment (LCA) experts and began adopting the method to utilize high resolution agronomical data covering livestock (primarily ruminants), grassland/forage productivity and quality, and arable systems established on its North Wyke Farm Platform (NWFP) and the LTEs. The NWFP is a UK ‘National Bioscience Research Infrastructure’ (NBRI) developed for informing and testing systems science utilising high‐resolution data to determine whether it is possible to produce nutritious food sustainably. Thanks largely to the multidisciplinary knowledge at RRes, and its collaborators, its LCA Team has been at the forefront of methodological advances during a 6‐year Institute Strategic Programme (ISP) ‘Soil‐to‐Nutrition’ (S2N). While S2N investigated the co‐benefits and trade‐offs of new mechanistic understanding of efficient nutrient use across scales from pot to landscape, this commentary specifically synthesizes progress in incorporating human nutrition in the context of environmental footprinting, known as ‘nutritional LCA’ (nLCA). We conclude our commentary with a brief discussion on future pathways of exploration and methodological developments covering various activities along entire agri‐food supply‐chains.

## 1 INTRODUCTION

Life cycle assessment (LCA) has been used for decades to identify pollution potential ‘hotspots’ and compare impacts to environmental health arising from various food systems (e.g. de Vries and de Boer, 2010). More recently, however, the LCA method has evolved to consider trade‐offs between environmental *and* human health using the ‘nutritional‐LCA’ (nLCA) approach (McAuliffe et al., 2020; McLaren et al., 2021). Rothamsted Research (RRes) is the world's oldest agricultural institute globally famous for its invention of the first commercially synthetic fertilizer (superphosphate) and long‐term farming experiments (LTEs), which provide open‐access data and information to inform optimal fertilizer rates in relation to various crop yields dating back over 170 years. In 2015, RRes established an LCA team tasked with: (a) utilizing high‐resolution (both spatially and temporally) data collected on research platforms at the institute to identify sustainable food systems capable of ensuring food security, and (b) advancing LCA using RRes's interdisciplinary expertise which forms part of the institute's uniqueness; for instance, RRes has in‐house modelling capabilities (often informed by high‐quality, primary data collected through targeted pot‐, plot‐ and field‐scale trials to assess pollutant mitigation measures' feasibility) to estimate farm geospatially heterogeneous farming typologies and interventions to reduce impacts to nature through exploration of interactions between soil health and environmental impacts. This commentary provides a brief synopsis of methodological progression regarding the LCA framework and novel environmental metrics developed as part of a 6‐year Institute Strategic Programme—Soil to Nutrition (S2N). S2N's funding comes to an end in March 2023, and therefore, this commentary focusses primarily on developments of novel metrics to explore the nexus between nutritional and environmental sciences, which RRes began its journey in the area through a publication in *Food and Energy Security* (*FES*; McAuliffe et al., 2018), utilising primary (and secondary) data provided directly through S2N experimental research and deep exploration of relevant literature.

## 2 NUTRITIONAL DENSITY SCORES

Human nutrient provision is often assessed at the food commodity level in the nutritional sciences using nutritional density scores (NDS), with perhaps the most widely adopted approach, certainly in an LCA context, being the Nutrient Rich Food (NRF9.3; Fulgoni et al., 2009) scoring system which assesses nine encouraged nutrients (i.e. protein, certain minerals and vitamins, and polyunsaturated fatty acids (PUFA)) and three nutrients (saturated fatty acids (SFA), sodium and added sugars) to be limited. The NRF9.3 framework assesses the benefit or risk of each nutrient in a food item against recommended daily intakes (RDI) for the population/geographic region under study. The approach results in a single score for each food, which can be positive or negative. While NRF9.3 is undoubtedly widely used in LCA, it has limitations: for example, unprocessed animal‐sourced foods do not contain fibre, a ‘nutrient’ considered under NRF9.3, making it an imperfect comparison for foods with notably different nutritional profiles (e.g. animal‐based produce vs. plant‐based produce). McAuliffe et al. (2018) identified this issue and began their nutrition‐environment nexus research journey in *FES* by developing a new country‐specific framework (UK Nutritional Index; UKNI) to compare animal‐based produce (species and production methodology) fairly and transparently. UKNI, inspired by work carried out in Finland (Saarinen et al., 2017), was subsequently used as a scaling factor, known in LCA as a ‘functional unit,’ to compare the environmental footprints of four meats, thus answering the question: ‘how much of a given meat would need to be consumed to meet the RDIs for a range of nutrients and what is the associated environmental footprint?’ The results indicated that less beef would need to be consumed to achieve the defined RDIs compared to the three other meats (lamb, chicken meat and pork); this was due primarily to the inclusion of long‐chain omega‐3 PUFA and zinc, which beef, particularly pasture‐produced beef, tends to have higher levels of compared to other meats. While this methodological development was merited at the time, there were limitations to the study such that foods are rarely eaten in isolation and therefore nutritional complementarity at the meal‐ or diet‐level should be explored, as will be discussed in Sections 4 and 5.

## 3 COMPLEXITY ASSESSMENT OF NUTRITIONAL LIFE CYCLE ASSESSMENT

**FIGURE 1:**
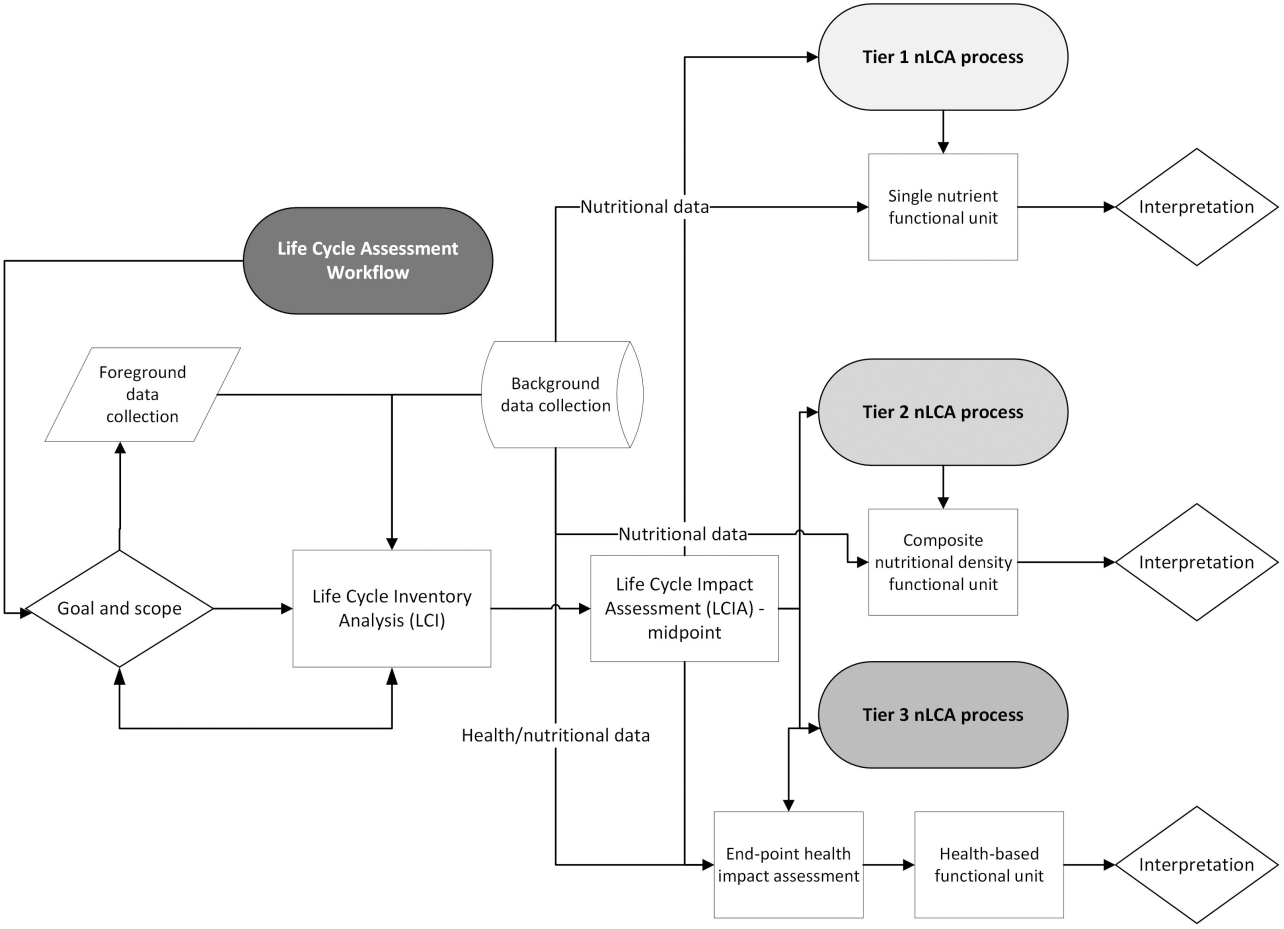
Workflow system diagram of how the various tiers of nLCA defined by McAuliffe et al. (2020) are generally conducted.

McAuliffe et al. (2020) carried out a literature review of nutritional LCA (nLCA) studies and developed a complexity level ranking system under three tiers (Figure 1). Tier 1 was defined as nLCAs, which consider one or multiple nutrients as functional units in isolation. Under Tier 1, protein was found to be the most used nutrient as a functional unit, but issues surrounding digestibility of protein (i.e. the anabolism of amino acids via absorption in the human gut) and quality (i.e. the composition and positioning of amino acids within the proteins quaternary structure) were acknowledged and subsequently addressed as will be described in Section 5. Tier 2, on the other hand, includes composite scoring systems as functional units (e.g. NDS as in McAuliffe et al., 2018). The second tier is most usually applied to single commodities, and many authors have developed their own NDS scoring systems as per McAuliffe et al. (2018) and explored in more detail in McAuliffe et al. (2020). Finally, Tier 3 typically develops novel ‘end‐point’ impact assessments (i.e. considering in tandem how environmental pollutants *and* nutritional profiles affect a commodity's impact on nature, for example biodiversity losses and gains, and human health, for example using disability‐adjusted life years, or DALY). This approach is inevitably the most complex tier under the nLCA framework, but it is worth noting that capturing uncertainties under this approach (and Tier 2, for that matter) is highly complicated and often overlooked, thereby leading to potentially misleading interpretation by consumers, stakeholders and policymakers in the context of food security and human health. RRes's LCA Team and global nutritional scientists are currently working towards highlighting these issues for future nLCA practitioners to be more aware of nutritional complexities while providing solutions to overcome said issues.

## 4 GLOBAL EXPERT REPORT ON NLCA BY NUTRITIONAL AND ENVIRONMENTAL SCIENTISTS

In 2021, the United Nations' Food and Agricultural Organization (FAO) commissioned a report to assess state‐of‐the‐art nLCA work holistically and identify strengths, weaknesses and gaps in knowledge which require further research (McLaren et al., 2021). RRes's role in the FAO's global assessment was on data provision, with a specific focus linking environmental footprint databases with nutritional composition databases. In this regard, the data‐based element of the report concluded that current combinations of the aforementioned databases lead to disjointed assessments as, for example the temporal and geographic boundaries of such databases may not align. Despite this arguably major limitation, nLCA experts are increasingly working with industry (e.g. farmers, retailers, distributors, etc.) to generate datasets which align environmental footprints with nutritional quantity and quality using primary data (e.g. see Lee et al., 2021 as an example of complexities related to primary data‐driven nLCA results from cradle to farmgate), thereby reducing the uncertainty of such analyses. In terms of nutritional *quality*, McLaren et al. (2021) highlighted that comparing nutritional *quantity* of food items is not robust enough to draw clinical conclusions (e.g. at the ‘end‐point’ (n)LCA level); to navigate this restriction, the authors recommended that complexities such as nutrient bioavailability and digestibility, both of which can be affected by ‘anti‐nutritional factors’ such as glucosinolates and tannins, particularly in plant‐based produce which restrict the uptake of certain nutrients including protein, should be considered. This is particularly imperative when comparing products which have different nutritional functions (e.g. sources of carbohydrates/energy, fibre, protein and water−/fat‐ soluble minerals and vitamins).

## 5 ASSESSING THE NUTRITIONAL QUALITY OF PROTEIN IN NLCA

As discussed in McAuliffe et al. (2020) and McLaren et al. (2021), protein content of a food tends to be the most commonly used functional unit under Tier 1 nLCA. However, protein anabolism is an incredibly complex process which depends on a balance of 21 proteinaceous amino acids in place and time; nine of which are solely sourced from the diet (indispensable amino acids (IAA) also referred to as essential amino acids) and the others, which although can be assimilated in situ, may become rate limiting. McAuliffe et al. (2023) drew upon findings reported in McLaren et al. (2021), which highlighted that protein *quality* should be incorporated into the nLCA framework when protein is being used as a functional unit. McAuliffe et al. (2023) used a protein quality assessment system known as Digestible Indispensable Amino Acid Score (DIAAS) to generate an ‘adjusted’ protein functional unit. The protein‐quality functional unit was applied to the carbon footprints (kg CO2‐eq/100 g protein) and land occupation (m^2^*year/100 g protein) of four animal‐based (dairy beef; cheese; eggs; pork) and four plant‐based (nuts; peas; tofu; wheat) products. The same analysis was carried using unadjusted protein as a functional unit. The study revealed that animal‐based products scored more than 100% (122%–141%) DIAAS due to their higher proportion of IAAs, highly digestible structure and lack of inhibitory compounds; tofu had the highest plant‐based DIAAS (105%), while the three other plant‐based protein sources scored under 100%, with wheat scoring particularly poorly (43%). This led to dairy beef's (DIAAS = ~140%) environmental footprints reduced substantially (~29%) under the adjusted protein functional unit. On the other hand, due to wheat's low DIAAS, its environmental footprints were increased by a factor of 2.3. McAuliffe et al. (2023), however, urged caution related to their novel approach to protein‐based functional units. This was due to the fact that, when consumed as part of a meal (or diet), IAAs can be balanced by combining low DIAAS foods with high DIAAS foods to promote protein anabolism through IAA complementarity, emphasizing the importance of balance between contrasting food groups, that is animal and plant‐sourced foods.

## 6 FUTURE DIRECTIONS FOR (N)LCA TO IMPROVE FOOD SECURITY AND METHODOLOGICAL RIGOUR

### 6.1 Inclusion of carbon stock changes in LCAs

Globally, soil organic carbon (SOC) accumulation as well as carbon uptake in plants (including trees and hedgerows) on agricultural land is expected to hold major potential to mitigate land‐based greenhouse gas (GHG) emissions (Petersen et al., 2013). However, there is a lack of reported impacts concerning SOC changes in LCAs of agricultural products (Jebari et al., 2022). This suggests that LCA practitioners may not have a well‐defined procedure to account for soil C in their assessments, despite it being a highly debated topic among sustainability experts. The evidence and impacts of C stock changes on LCA may differ among various agricultural products and management practices. For instance, in the case of dairy products, a major contributor to GHG emissions in the agricultural sector, including C stock changes has been shown to reduce the global warming potential of European dairy products by 9% of the overall GHG emissions in moist temperate Spanish grasslands associated with dairy production (Jebari et al., 2022). Regardless of whether being applied to environmental LCA or nLCA, more robust assessments of food supply‐chains using dynamic carbon models, such as RothC (Nemo‐Klumpp et al., 2017), will have implications for interpretation of (n)LCA results. RRes is currently addressing this gap in knowledge using primary data from the NWFP.

### 6.2 Applying nutritional science to LCAs of rotational systems producing multiple co‐products

On‐going work at the National Agricultural Research Institute of Uruguay (INIA) in collaboration with RRes has been assembling high‐resolution data, including carbon stocks, crop yields, soil quality and animal performance in no‐till rotational systems which produce multiple co‐products. For example, INIA's Palo a Pique Long‐Term Experiment was installed in 1995, where the main objective was evaluating no‐till technology in four rotational systems under direct grazing in soils with severe limitations (e.g. erosion and degradation risk and poor soil drainage). These systems produce multiple products (e.g. beef, wheat, oat and soybean) both on an annual basis and cross‐year basis, depending on the system under investigation. This provides ample opportunities to advance (n)LCA by considering nutritional provision from rotational agricultural systems, an understudied aspect in terms of agri‐food sustainability and food security, which produce multiple food products (e.g. Shrestha et al., 2020). For example, nutritional metrics can be applied to the four systems trialed at Palo a Pique (each of which provides different agricultural products) to determine which system produces the most nutritionally‐ and environmentally friendly outputs at a land‐use level rather than at an individual product level, e.g. beef or soybean.

### 6.3 Food waste and implications of reducing losses throughout entire supply‐chains

Food waste occurs at various stages in the food supply chain (e.g. production, processing, transportation and consumption), with maximum losses (70%) at consumption (e.g. households, restaurants and supermarkets. Therefore, it is essential to consider agri‐food systems beyond the farmgate including food losses to avoid environmental impacts from food production which does not get consumed. Food waste can be managed through various means; for instance (in no particular order): composting, anaerobic digestion, incineration, donation to food banks, animal feed production and landfilling ideally with landfill gas utilization, are all promising options for further exploration. Due to the heterogeneity in the characteristics and composition of food waste generated at retail and consumer stage, a region‐specific (e.g. national scale) LCA study is essential to evaluate the environmental footprints of food waste and its implications on food security and nutritional provision. Indeed, it is evident that a lot of gaps are available which can be filled with LCA studies beyond the farmgate to reduce the overall environmental impacts of the food supply chain. The availability of reliable foreground and background data is the most critical part of LCA studies. RRes has been extensively working at the farm‐scale (i.e. cradle to farmgate) and providing scientific communities, government and farmers with scaled‐up and fit‐for‐purpose sustainability solutions for UK food production. However, in progressing, RRes has acknowledged that nLCA research needs to cover the entire food supply chain and, as a result, has built an LCA team with expertise beyond the farmgate. Future research will consider nutritional implications of food waste for major food commodities consumed within the UK.

### 6.4 Future directions for nLCA

In addition to the novel areas of research identified above, RRes is also working alongside global nutritional experts to improve the scientific rigor of nLCA, covering all tiers defined by McAuliffe et al. (2020). For example, as mentioned in Section 5, McAuliffe et al. (2023) provided a simple yet informative case study to build upon in terms of incorporating digestibility and bioavailability of various food items and their nutritional composition into the nLCA framework. Further, on‐going work is assessing the complementarity of quality‐adjusted metrics for broader nutrients than protein at the meal‐ and diet‐level. Lastly, nLCA has, to date, focused on the intersection between food security and environmental impacts. Future research streams are exploring the nexus between nutritional provision and societal and economic impacts beyond human health including, for example, rural economies, human and animal welfare and food production displacement. Despite its current limitations, nLCA is a promising tool for informing policymaking in terms of delivering equitable, environmentally friendly and healthy food systems across the globe. However, more scientifically robust primary data (i.e. sourced from industry) and interpretation of results (e.g. uncertainty and sensitivity analyses) require urgent attention and methodological development.

## CONFLICT OF INTEREST STATEMENT

The authors have stated explicitly that there are no conflicts of interest in connection with this article.

## Data Availability

All underlying data discussed and summarised in this commentary are available from the individual papers covered herein. Rothamsted Research has an Open Access policy following UKRI mandates; as a result, any data (with the exception of commercially or personally sensitive information) utilised or generated through the RRes LCA Team and collaborators is always made publically available through data repositories and/or data publications. The authors will be happy to provide further information upon request, if necessary.
